# Complete Remission of Recurrent Primary Cutaneous Anaplastic Large Cell Lymphoma Following Bexarotene Therapy: A Case Report

**DOI:** 10.7759/cureus.94700

**Published:** 2025-10-16

**Authors:** Natsumi Shimada, Natsuko Saito-Sasaki, Hitomi Fujimura, Hikaru Kawahara, Etsuko Okada, Yu Sawada

**Affiliations:** 1 Dermatology, University of Occupational and Environmental Health, Kitakyushu, JPN

**Keywords:** bexarotene, case report, complete remission, primary cutaneous anaplastic large cell lymphoma, recurrent

## Abstract

Primary cutaneous anaplastic large cell lymphoma (pcALCL) is a CD30-positive cutaneous T-cell lymphoma with a generally favorable prognosis. While radiotherapy is effective, recurrence is not uncommon. Herein, we report the case of an 87-year-old man with recurrent pcALCL who achieved complete remission after bexarotene treatment, despite resistance to radiotherapy and topical steroids. This case highlights bexarotene as a promising therapeutic option for refractory pcALCL. Our experience reinforces the potential of bexarotene as a viable treatment for pcALCL, particularly in recurrent or refractory cases where conventional therapies are contraindicated or ineffective.

## Introduction

Primary cutaneous anaplastic large cell lymphoma (pcALCL) is characterized by CD30 expression in more than 75% of tumor cells and is considered part of the spectrum of CD30-positive cutaneous lymphoproliferative disorders [1], in contrast to systemic ALCL, which often presents with nodal or visceral disease. PcALCL generally has a favorable prognosis but may relapse locally. Although pcALCL has a favorable prognosis, with a five-year survival rate exceeding 90%, recurrence can occur in up to 40% of cases following standard therapies such as radiotherapy [2]. Bexarotene, a synthetic retinoid, has shown efficacy in cutaneous T-cell lymphomas [3]. Common adverse effects of bexarotene include hypertriglyceridemia, hypothyroidism, and hepatotoxicity; therefore, regular monitoring of lipid levels, thyroid function, and liver enzymes is recommended during treatment. Reports of its successful use in pcALCL remain limited. We report a refractory case of pcALCL successfully treated with bexarotene.

## Case presentation

**Figure 1:**
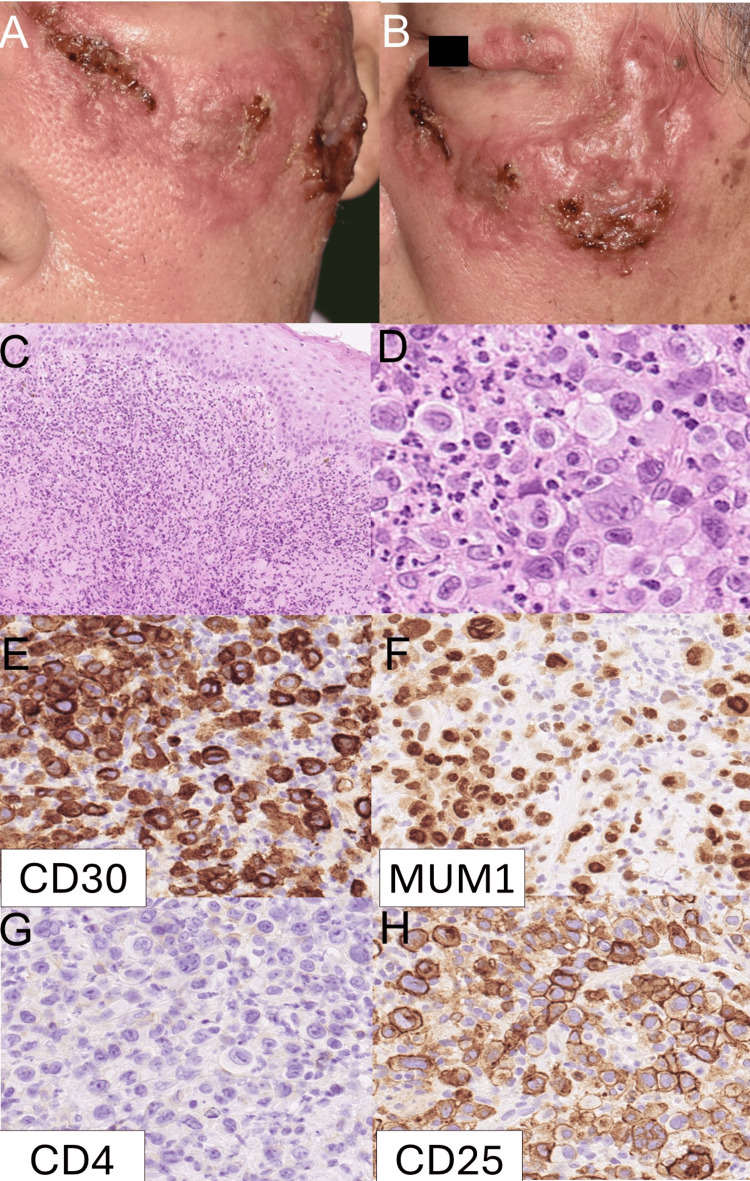
Initial clinical, histological, and immunohistochemical findings. (A, B) Clinical photographs at initial presentation showing a reddish tumor on the left lower eyelid. (C, D) Hematoxylin and eosin (HE) staining revealing large atypical lymphoid cells with pleomorphic nuclei. (E–H) Immunohistochemical staining of the tumor cells showing positivity for CD30 (E), CD4 (F), MUM1 (G), and CD25 (H), supporting the diagnosis of pcALCL.

An 87-year-old man presented with a reddish tumor on his left lower eyelid. Thirteen years earlier, he had been diagnosed with pcALCL on his left cheek and forehead and had achieved complete remission following IFN-γ therapy. At the time of re-presentation, physical examination revealed a solitary erythematous nodule on the left lower eyelid. The lesion was firm, mildly tender, and measured approximately 1.5 cm in diameter. The patient reported no associated pain or systemic symptoms. The lesion rapidly enlarged over the following weeks (Figure 1A, B). A skin biopsy showed large atypical lymphoid cells that were CD30+, CD4+, MUM1+, and CD25+ (Figure 1C-F). The diagnosis was supported by histological features and immunohistochemical markers, including diffuse CD30 positivity in large atypical lymphoid cells and absence of ALK expression, consistent with a diagnosis of pcALCL (T2aN0M0).

**Figure 2:**
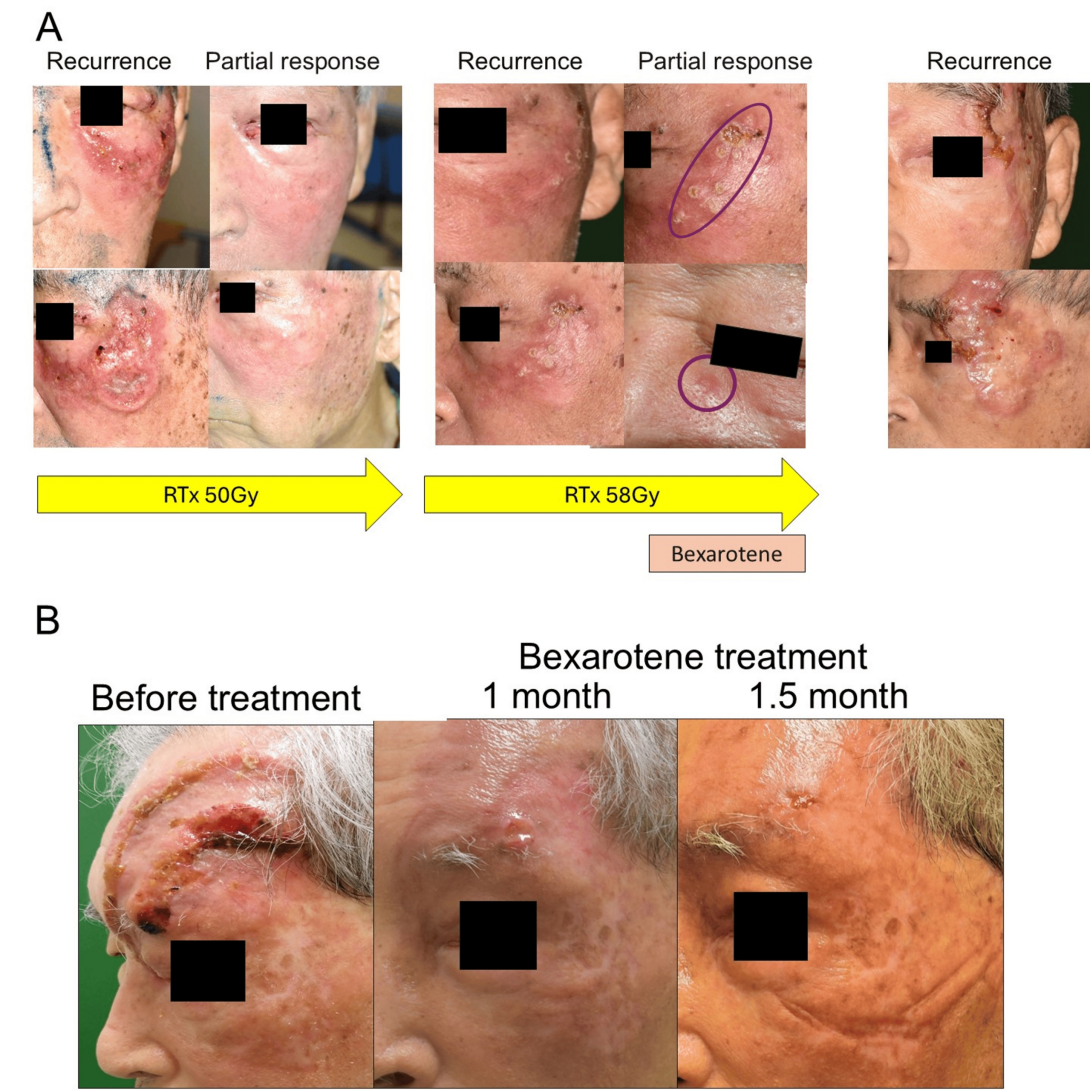
Clinical course before and after bexarotene treatment. (A) Serial clinical photographs demonstrating the progression and recurrence of the lesion despite corticosteroid therapy and radiotherapy (50 Gy and 58 Gy). (B) Marked tumor regression and complete remission achieved 1.5 months after initiating oral bexarotene.

Radiotherapy was initiated with a total dose of 50 Gy; however, the lesion recurred within two weeks after completion (Figure 2A). Despite additional radiotherapy, the tumor relapsed repeatedly, ultimately requiring a cumulative dose of 108 Gy. In light of the treatment resistance, oral bexarotene (525 mg/day) was initiated. The treatment was temporarily interrupted for surgical resection of newly diagnosed colon cancer, during which the tumor showed rapid growth. Given the progression and prior reports of efficacy in cutaneous T-cell lymphomas, oral bexarotene was re-administered. Within weeks, tumor regression was evident, and the lesion continued to shrink steadily. By 1.5 months after treatment initiation, the patient achieved complete clinical remission with no apparent adverse effects related to bexarotene (Figure 2B). The response was assessed by clinical observation and serial photographs, confirming sustained remission for more than six months. Routine laboratory monitoring, including lipid profile and liver function tests, revealed no abnormalities throughout the treatment period.

## Discussion

pcALCL is a CD30-positive lymphoproliferative disorder characterized by solitary or localized nodules and tumors, typically showing indolent behavior and a favorable prognosis [4]. Radiotherapy is generally considered one of the first-line treatments for localized disease [5]. In the present case, the patient initially achieved partial remission following radiotherapy but experienced rapid local recurrence shortly thereafter. Despite repeated courses of radiation, the lesion relapsed multiple times, ultimately requiring a cumulative dose of 108 Gy. Given the patient’s advanced age and multiple comorbidities, systemic chemotherapy was deemed high risk.

Bexarotene, a selective retinoid X receptor (RXR) agonist, is approved for cutaneous T-cell lymphomas and modulates gene expression related to differentiation and apoptosis [6]. Although its role in pcALCL is not yet well established, several case reports have indicated potential efficacy in selected cases. In our case, bexarotene was introduced as a systemic treatment. Treatment was temporarily suspended due to the discovery and surgical resection of newly diagnosed colon cancer, during which time the tumor rapidly regrew. Reinitiation of bexarotene led to marked and sustained clinical remission without notable adverse effects.

Several case reports have described the successful use of bexarotene in patients with ALCL, as summarized in Table 1 [7-9]. Complete remission was achieved in all reported cases, including those treated after failure of chemotherapy or corticosteroids. Notably, our case represents the oldest reported patient (87 years) and is unique in that the lesion demonstrated resistance to high-dose radiotherapy. Moreover, this case is distinguished by the clear temporal correlation between reinitiation of bexarotene and prompt, sustained tumor regression.

**Table 1:** Summary of reported cases treated with bexarotene for ALCL. ALCL: anaplastic large cell lymphoma, CHOP: cyclophosphamide, doxorubicin, vincristine, and prednisone, CR: complete remission.

Authors	Age	Gender	Site	Previous treatment	Bexarotene dose	Outcome
Sheehy et al. [7]	40	Female	Face, abdomen, thigh	CHOP chemotherapy	525 mg	CR
Keun et al. [8]	80	Female	Hand, forearm, leg	Prednisolone, cyclophosphamide, dexamethasone, electrobeam	300 mg/m^2^	CR
Ardigò et al. [9]	74	Male	Face, trunk, extremities	Multiple chemotherapy	300 mg/m^2^	CR
Our case	87	Male	Face	Radiation	300 mg/m² → 150 mg/m²	CR

These findings suggest that bexarotene may exert meaningful antitumor activity in pcALCL, even in previously irradiated or treatment-resistant lesions. The ability to induce durable remission with an oral agent that is generally well tolerated makes bexarotene particularly appealing for elderly or frail patients in whom aggressive systemic chemotherapy is not feasible.

Mechanistically, bexarotene is thought to promote tumor cell differentiation and apoptosis via RXR-mediated transcriptional regulation [10]. Although its effects have been well characterized in mycosis fungoides and Sézary syndrome [11], its application in pcALCL remains underrecognized. This case contributes to the limited but growing body of evidence supporting its use in this subtype.

## Conclusions

In summary, our experience reinforces the potential of bexarotene as a viable treatment option for pcALCL, particularly in recurrent or refractory cases where conventional therapies are contraindicated or ineffective. Further accumulation of clinical data is needed to better define its optimal use and long-term outcomes in this context. Although our patient was elderly and refractory to radiotherapy, bexarotene may also be considered for patients with recurrent or multifocal pcALCL, or for those in whom conventional therapies are contraindicated because of comorbidities or cumulative radiation exposure. However, as this report describes a single case, larger prospective studies are warranted to confirm the efficacy and safety of bexarotene in a broader patient population.
